# Acaricide Resistance Monitoring and Structural Insights for Precision *Tetranychus urticae* Management

**DOI:** 10.3390/insects16050440

**Published:** 2025-04-23

**Authors:** Said Kewedar, Qi-Ren Chen, Timothy W. Moural, Carah Lo, Elsie Umbel, Peter J. Forrence, Douglas B. Walsh, Fang Zhu

**Affiliations:** 1 Department of Entomology, Penn State University, University Park, PA 16802, USA; saeed.kewedar@gmail.com (S.K.); qfc5054@psu.edu (Q.-R.C.); twm78@psu.edu (T.W.M.); carahlo@yahoo.com (C.L.); emumbel42@gmail.com (E.U.); 2 Irrigated Agriculture Research and Extension Center, Washington State University, Prosser, WA 99350, USA; peter.forrence@wsu.edu (P.J.F.); dwalsh@wsu.edu (D.B.W.); 3 Department of Entomology, Washington State University, Pullman, WA 99164, USA; 4 Huck Institutes of the Life Sciences, Penn State University, University Park, PA 16802, USA

**Keywords:** acaricides, target site insensitivity, molecular markers, mutations, protein structure, molecular docking

## Abstract

The two-spotted spider mite (*Tetranychus urticae*) is a highly destructive and economically significant pest in agricultural, horticultural, and ornamental agroecosystems worldwide, including hop (*Humulus lupulus*) and mint (*Mentha* spp.) fields in the Pacific Northwest (PNW) region of the United States. Repeated acaricide applications and rotations have led to widespread resistance, resulting in control failures. In this study, we investigated the mechanisms of resistance to four different acaricides (bifenthrin, bifenazate, etoxazole, and abamectin) across 23 field-collected TSSM populations by integrating diagnostic bioassays, genetic screening for resistance-associated mutations, structural modeling, and molecular docking. Several *kdr* mutations and mutation combinations were detected in *TuVGSC* across all tested populations. The G132A in *Tucytb* was identified in 68.75% of hop and 40% of mint TSSM populations, while the I1017F in *TuCHS 1* was found in 94% of hop and 100% of mint populations. Structural analysis revealed key interactions between acaricides and target proteins in both wild-type and mutant variants, providing novel insights into the functional impacts of these mutations. Our findings enhance the understanding of TSSM adaptation to acaricides among different crops, supporting the development of more effective resistance management strategies to mitigate economic losses in hops, mint, and other crop production.

## Simple Summary

The two-spotted spider mite (*Tetranychus urticae*) is a significant economic pest that feeds on a wide range of plants, including economically important crops such as hops and mint. Farmers commonly use chemical treatments (acaricides) to control infestations. However, over time, these mites have developed resistance, reducing the effectiveness of these treatments. In this study, we evaluated resistance levels and investigated the underlying genetic mechanisms of resistance in *T. urticae* populations collected from United States Pacific Northwest hopyards and mint fields. By examining 23 mite populations, we identified specific genetic mutations associated with resistance and analyzed how these mutations influence the interaction between target proteins and acaricides. Our findings provide valuable insights into the molecular basis of resistance and offer multiple perspectives for improving pest control strategies. This research supports the development of more effective and sustainable management approaches, helping farmers and agricultural professionals mitigate economic losses caused by this pervasive pest.

## 1. Introduction

*Tetranychus urticae* (commonly known as the two-spotted spider mite, TSSM) is a highly destructive pest that imposes a substantial drain on global agriculture. As an extremely polyphagous herbivore, the TSSM feeds on over 1100 plant species, including vital economic crops such as hops, mints, raspberries, cucumbers, tomatoes, peppers, strawberries, corn, apples, grapes, almonds, and various ornamental plants [1,2,3,4,5,6]. The TSSM attacks host plants by extracting cell contents, causing pale spots or scars. TSSM feeding activities cause plant cell disruption, reduce chlorophyll, and consequently impair photosynthesis, leading to significant losses or total crop failure [7,8]. Hops (*Humulus lupulus*), which are primarily grown in the Pacific Northwest (PNW) of the United States, are particularly vulnerable to the TSSM [9,10]. Hops are crucial for the brewing industry, providing the bitterness, flavor, and aroma that are integral to beer [9]. The U.S. hop industry is predominantly located in the State of Washington, which contributed 74% of the production, followed by Idaho with 16% and Oregon with 10%. Collectively, these states accounted for over 99% of U.S. hop acreage and 40% of global production in 2023 (National Hop Report 12/20/2023). The value of the hop industry in the United States was estimated at USD 562 million for the same year (USDA NASS). Similarly, mints, cultivated for their culinary and commercial uses, also face significant losses due to mite infestations and subsequent feeding damage [6]. Peppermint (*Mentha piperita* L.) and spearmint (*Mentha spicata* L.) are perennial specialty crops primarily cultivated for their oil, which is used in various products, including flavoring agents for condiments, chewing gum, and dental and medical products [11]. The PNW produces approximately 90% of the peppermint oil in the United States, with Idaho and Washington being the leading producers. In 2023, peppermint and spearmint production in Washington was valued at USD 33.5 million (USDA NASS). In the PNW, the warm and dry summer climate makes the TSSM the dominant arthropod pest, significantly impacting both the quantity and quality of hop and mint production [6,9,11].

Sustainable TSSM management in agroecosystems involves the use of biological control reagents, cultural control, population monitoring, and chemical control [1,4,12]. To prevent mite populations from reaching damaging economic thresholds, various acaricides with different modes of action are used in rotation or combination during the growing season [9,10]. These acaricides include chemicals targeting neuron proteins, such as sodium channel modulator pyrethroids (IRAC group 3A); glutamate-gated chloride channel (GluCl) allosteric modulators (IRAC group 6); chemicals targeting energy metabolism, such as the Mitochondrial Electron Transport Inhibitors (METIs, IRAC groups 20, 21, 24, and 25); and chemicals targeting proteins associated with growth and development, such as mite growth inhibitors (IRAC group 10) [13,14]. For example, bifenthrin targets the voltage-gated sodium channel of mites (TuVGSC) and is favored for its long residual activity, low cost, and safety. Bifenazate targets mitochondrial complex III (cytochrome b, Tucytb) and is valued for its rapid knockdown and selectivity for pests over beneficial insects. Etoxazole, a mite growth inhibitor (MGI), disrupts chitin synthesis by targeting the chitin synthase 1 (TuCHS1) in TSSM [4,6]. Abamectin, an avermectin-class pesticide targeting the glutamate-gated chloride channel of mites (TuGluCl), was initially developed for veterinary use and later adapted for agricultural pest control [9]. Grower records indicate that in some years, as many as nine different acaricides are applied during a single hop growing season in the PNW [9,10]. However, the TSSM holds the notorious distinction of being the world’s second most resistant arthropod, following the diamondback moth (*Plutella xylostella*), with a documented resistance to 96 unique insecticide and acaricide active ingredients across 263 locations globally (ARPD; available at http://www.pesticideresistance.org [20 January 2025]). This remarkable ability to develop resistance is exacerbated by both its biology and agricultural practices. Frequent acaricide applications accelerate resistance development, while biological traits such as high fecundity (more than 100 eggs per female), a short generation time (approximately 10 days at 25 °C), and arrhenotokous reproduction facilitate the rapid selection of resistance genes [3]. As a result, farmers often experience control failures, as the efficacy of acaricides is severely limited over time [9]. Resistance development is a complex process influenced by multiple mechanisms [15,16,17]. Like insects, the TSSM can develop resistance through behavioral adaptations, reduced penetration, enhanced sequestration, metabolic detoxification, or target site insensitivity [8,13,18,19,20]. Recent reviews identified over 25 mutations associated with various forms of acaricide resistance, many of which can serve as molecular markers for field-based resistance monitoring [21]. Structural analyses examining how acaricides interact with their target proteins carrying specific mutations identified in TSSM populations offer a powerful approach to advancing Insecticide Resistance Management [22,23].

This study aims to conduct a comprehensive diagnostic analysis of pesticide resistance in TSSM populations collected from hopyards and mint fields in the PNW of the U.S. We assessed the resistance status of TSSM populations to two commonly used acaricides, bifenazate and abamectin, through bioassays. Next, we screened mutations associated with resistance to four different acaricides (bifenthrin, bifenazate, etoxazole, and abamectin) across 23 field-collected TSSM populations using molecular techniques. Furthermore, molecular docking techniques were utilized to elucidate how these acaricides interact with proteins harboring resistance-associated mutations in TSSMs. Our goal was to model the binding interactions between acaricides and mutated proteins to better understand how genetic changes affect acaricide resistance efficacy at the structural level. These findings will inform the development of more effective, sustainable pest management practices, contributing to the long-term health of agricultural systems.

## 2. Materials and Methods

### 2.1. Mite Populations

**Figure 1:**
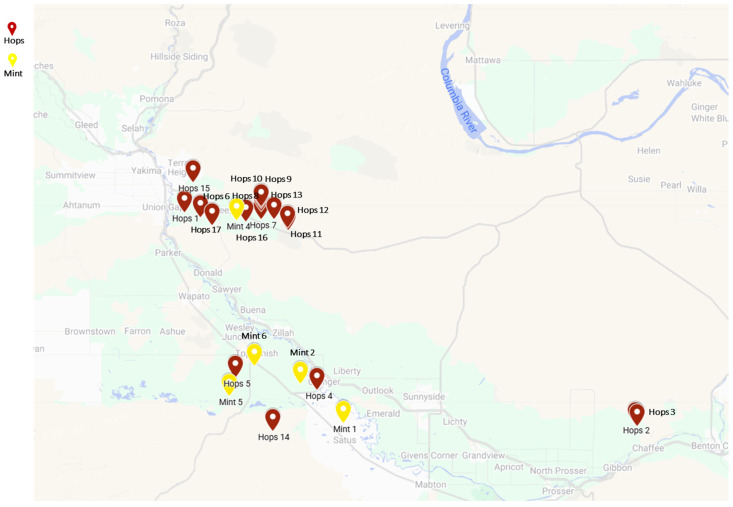
The geographic distribution of the TSSM populations collected from hops (Ph) (Hops, red color) and mint (Pm) (Mint, yellow color). Google Maps (accessed on 10 August 2024) was used to pinpoint the collection sites for each sample.

Twenty-three populations of the two-spotted spider mite were collected from commercial hopyards and mint fields in the Yakima Valley, WA, USA between October 2022 and October 2023. Our naïve acaricide-susceptible population has been reared on lima bean plants since its original collection from feral grape vines in Montana, U.S., in 1995. Ever since, this population has not been exposed to any acaricides or insecticides [5,9]. The specific locations are illustrated in Figure 1 and with detailed information in Table S1. Hop leaves infested with mites were collected, placed in plastic bags, and stored in ice coolers. The samples were transported to the lab within a few hours. Spider mites were identified under a dissecting scope based on morphological characteristics. The mites were reared on 2-week-old lima bean plants (*Phaseolus lunatus* L.) at 28 ± 2 °C, 70 ± 5 RH, and a photoperiod of 16:8 (L:D) h in an isolated walk-in growth chamber at the Irrigated Agricultural Research and Extension Center (IAREC) in Prosser, WA, for one generation and then used for subsequent experiments. Approximately 200 adults were preserved in 95% ethanol for gDNA extraction.

### 2.2. Bioassays

Female adult mites were submitted to leaf disc bioassays with two acaricides, bifenazate and abamectin, following the method used in our previous studies [9]. In brief, we prepared leaf disc bioassay arenas using a clean Petri dish (9 cm diameter, 1.5 cm height; Alkali Scientific, Pompano Beach, FL, USA), into which we placed cotton (4 cm × 4 cm) soaked with clean water to ensure that the mites could not escape without drowning. Leaf discs, 2 cm in diameter, were then cut from 2-week-old lima bean plants and positioned on top of the cotton. To prevent the mites from escaping, clean forceps were used to gently pull the cotton around the edges of each leaf disc, forming a wall. Using a clean size-20 paintbrush, we carefully transferred at least 10 gravid adult female spider mites onto each leaf disc. Two commercially formulated acaricides for leaf disc bioassay are Epi-mek^®^ 0.15 EC (2% a.i. Abamectin, Syngenta Crop Protection, Basel, Switzerland) and Acramite^®^ 50WS (50% a.i. Bifenazate, Chemtura Agro Solutions, Philadelphia, PA, USA). A Potter precision spray tower was then used to apply 2 mL of each concentration of the acaricide to the leaf discs. The concentrations of acaricide ranged from 0 (control with distilled Millipore-filtered water only) to the field-recommended dose for hops [9]. The field-recommended doses for abamectin and bifenazate are 22.5 and 224 mg a.i./L, respectively [9]. The Potter spray tower was calibrated to deliver 2.0 ± 0.1 mg/cm^2^ of liquid under 1.1 kg/cm^2^ of pressure. After 24 h, the number of live and dead mites was counted. Where possible, we calculated the LC_50_ (the lethal concentration required to kill 50% of the population), along with the 95% confidence interval, the slope of the dose–response curve, Chi-squared values, and degrees of freedom using Minitab software https://www.minitab.com/en-us/ (State College, PA, USA; accessed on 10 August 2024). A resistance ratio was calculated, where applicable, by dividing the LC_50_ values of field samples by the LC_50_ value of the lab susceptible population.

### 2.3. Detection of Mutations Associated with Acaricide Resistance

Genomic DNA (gDNA) was extracted from about 200 adult mites from each of the susceptible and 23 field-collected TSSM populations using the Monarch^®^ HMW DNA Extraction Kit (New England Biolabs, Ipswich, MA, USA). The quality and quantity of gDNA were measured using a spectrophotometer (NanoDrop One, Thermo Scientific, Wilmington, DE, USA). DNA samples with an A260/A280 value of ~1.8 were used for PCR. The gDNA was stored at −20 °C till use. We analyzed mutations associated with resistance to 6 acaricides. The acaricide and mutation information, as well as relevant references, are shown in Table S2. The PCR was performed in a Bio-Rad T110 Thermal Cycler (Bio-Rad Inc., Hercules, CA, USA). Each PCR reaction contained 1–2 µL of gDNA (~100 ng/µL), 5 µL of PCR buffer, 0.8 µL of dNTP mix (10 mM), 0.6 µL of forward and reverse primers (Table S3), 1 µL of Phusion High-Fidelity DNA Polymerase (Thermo Scientific, Pittsburgh, PA, USA), and 11.6–12.6 of µL ddH_2_O. PCR was conducted under the following cycling parameters: 95 °C for 3 min and 50 s, 35 cycles of 94 °C for 35 s, 45–55 °C for 35–45 s, and 72 °C for 1–3 min, with a final extension for 10 min at 72 °C. Finally, 2 µL of PCR product was run on a 1% agarose gel and evaluated for integrity under UV light. PCR products were sequenced in Functional Biosciences Inc. (Madison, WI, USA). The sequence results were analyzed in SnapGene Viewer 7.2.1 (GSL Biotech LLC, Boston, MA, USA).

### 2.4. AlphaFold 2 Models and Molecular Docking for Acaricides

The protein models for TuVGSC, Tucytb, and TuCHS1 were predicted using ColabFold v1.5.5: AlphaFold2 using MMseqs2 [24,25]. The settings changed from default, the number of relaxations was set to 1, template mode was set to pdb100, the number of recycles was set to 12, max_msa was set to 32:64, and num_seeds was set to 2. The full-length sequences were used for predictions, and low-confidence regions based on pLDDT scores below 70 of the resulting AlphaFold 2 models were removed. For molecular docking, pesticide compound structures were retrieved from PubChem [26]. Molecular docking was performed using AutoDock Vina implemented in the DockingPie 1.2 plugin installed in PyMOL 3.0.4 with its exhaustiveness set to 20, possible poses increased to 10, and grid box set to cover the protein [27,28,29,30]. Additionally, binding cavities were calculated with the CavitOmiX plugin, using the default settings [31]. For TuVGSC, membrane localization was predicted using the PPM 3.0 server [32]. PoseVeiw was used to generate a ligand interaction diagram [33,34,35,36]. Model analysis was carried out with Chimera, ChimeraX, and PyMOL. Resulting figures were rendered with ChimerX v1.8 [37,38,39].

## 3. Results

### 3.1. Different Resistance Patterns of TSSMs Collected from Hop and Mint Fields

Our study assessed the acaricide resistance levels of TSSM populations collected from hopyards in Yakima Valley, WA, USA, during 2022 and 2023. Potter precision tower bioassays were performed to evaluate resistance to bifenazate and abamectin across different TSSM populations. As shown in Table S4, all TSSM populations tested showed moderate to high levels of resistance to abamectin and bifenazate. The LC_50_s in TSSM populations ranged from 11.110 mg a.i./L to 1694.300 mg a.i./L of abamectin. Populations such as Ph-1, Ph-3, and Ph-4 displayed significantly higher LC_50_ values, indicating a notable increase in resistance. For instance, Ph-1 exhibited an LC_50_ as high as 1694.300 mg a.i./L (RR > 2000 fold). The LC_50_s in TSSM populations ranged from 14.385 mg a.i./L to 260.843 mg a.i./L of bifenazate, with the highest LC_50_s in Ph-15 and Ph-16 being 168.111 mg a.i./L (RR = 205.01-fold) and 260.843 mg a.i./L (RR = 318.10-fold), respectively (Table S4).

**Figure 2:**
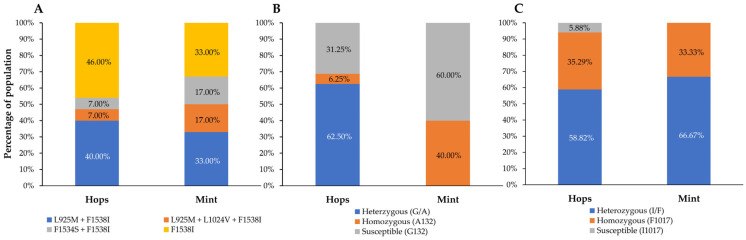
Different resistance patterns of TSSM populations collected from hop and mint fields. (**A**) *kdr* mutation combinations; (**B**) G132A mutation on *Tucytb*; (**C**) I1017F mutation on *TuCHS1*.

We also screened and analyzed mutations associated with resistance to various acaricides (Table S2). Among the 23 mutations on five target genes investigated, 4 *kdr* mutations (L925M, L1024V, F1534S, and F1538I) (Table 1), the G132A mutation on Tucytb, and the I1017F on *TuCHS 1* (Table 2) were identified. No mutations associated with abamectin were detected. The F1538I mutation alone was detected in 46% and 33% of the TSSM populations collected from hop and mint fields, respectively (Figure 2). The rest of the populations tested showed *kdr* mutation combinations, including L925M + F1538I, L925M + L1024V + F1538I, and F1534S + F1538I (Figure 2, Table 1). The L925M + F1538I combination was identified in 40% of hop TSSM populations and 33% of mint TSSM populations (Figure 2, Table 1).

Our results showed the genetic variation of G132A on *Tucytb* and I1017F on *TuCHS 1* in TSSM populations collected from hop and mint fields. The key mutations analyzed for *Tucytb* include G126S, G132A, A133T, I136T, S141F, L258F, D161G, and P262T. Only the G132A mutation was detected in 68.75% of TSSM populations collected from hopyards, while 40% of TSSM populations collected from mint fields harbored this mutation (Figure 2, Table 2). Most hop TSSM populations (10 out of 16) harbored a heterozygous mutation of G132A, while the homozygous mutation A132 was only detected in 1 population. However, in mint TSSM populations, two out of five populations tested harbored the homozygous mutation A132 (Figure 2, Table 2). Regarding the I1017F mutation in TuCHS 1, it was detected in almost all populations except one hop TSSM population. The homozygous mutation F1017 was identified in 6 out of 17 hop TSSM populations and two out of six mint TSSM populations (Figure 2, Table 2).

### 3.2. kdr Mutations Associated with Pyrethroid Resistance in Hop and Mint TSSM Populations

**Figure 3:**
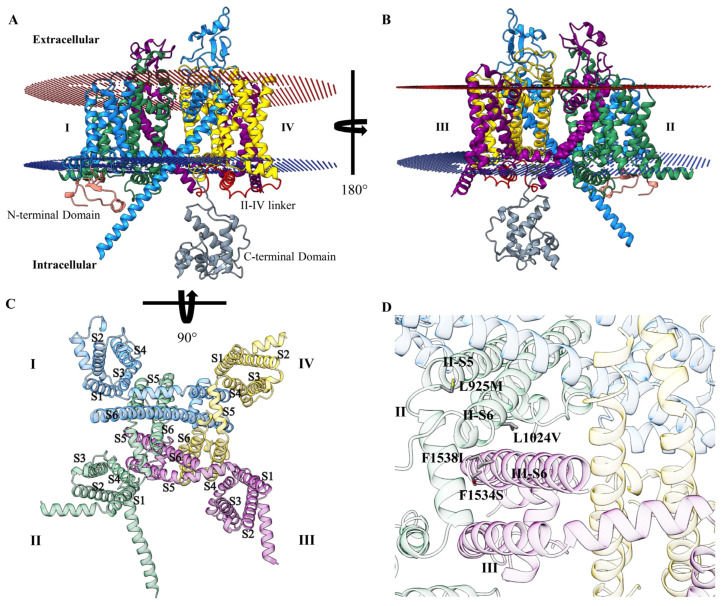
TuVGSC predicted 3D structure. (**A**) Localization in the cell membrane with extracellular and intracellular locations labeled. Ribbons have been colored by domain, with N-terminal domain colored salmon, domain I colored in blue, domain II in green, domain III in purple, domains III–IV linker in red, domain IV in yellow, and C-terminal domain in gray. (**B**) Rotated with respect to (**A**) 180° about the *y*-axis to show the respective localizations of D I, D II, D III, and D IV in the cell membrane. (**C**) TuVGSC highlighting the pore region, looking from the inside to the outside of the cell. Domains I through IV are labeled with their respective six helical segments, labeled S1 through S6. (**D**) Zoomed-in view of the TSSM VGSC pore region with mutations L925M and L1024V shown on S5 and S6 of domain II. Additionally, mutations F1534S and F1538I are shown on S6 of domain III. Residue mutations are numbers based on sequence alignment of *Musca domestica* VGSD (NCBI accession: AAB47604).

The 3-dimensional structure of TSSM voltage-gated sodium channel protein (TuVGSC) was predicted based on its amino acid sequence (NCBI accession: AFU35097) using ColabFold v1.5.5: AlphaFold2 using MMseqs2 [24,25]. The TuVGSC protein model consisted of three regions: the extracellular, transmembrane, and intracellular regions (Figure 3). Among these regions, the intracellular (cytoplasmic) region exhibited the lowest modeling confidence scores based on pLDDT local confidence scoring, and as a result, disordered loops were trimmed from the model. The extracellular region had pLDDT scores ranging mainly from 70 to 90, and thus, the backbone was deemed well modeled. Of the three regions, the transmembrane region was modeled with the highest pLDDT scores, mainly consisting of scores in the high- to very high-confidence range. The closest experimentally determined structural homologue to TuVGSC was the human voltage-gated sodium channel Nav1.2 (PDB ID: 6J8E), a cryo-EM structure, which shares 52% sequence identity with TuVGSC [40]. Additionally, the VGSC structure from an insect, specifically the American cockroach (NavPaS), is available (PDB IDs: 5X0M and 6A90) [41,42]. The trimmed TuVGSC model was superimposed onto NaV 1.2 and NavPaS, resulting in Cα RMSD values of 1.314 Å and 1.997 Å, respectively. Based on the high pLDDT scores and favorable RMSD values relative to experimentally solved homologues, the predicted model is of good quality and suitable for structural analysis. The transmembrane region of TuVGSC consisted of four domains: I, II, III, and IV (Figure 3A,B). Each domain contains six helical segments (S1 to S6) that span the membrane. The organization of the transmembrane domains and the structural layout of S1 through S6 in each domain are illustrated in Figure 3C. In each domain, segments S1 to S4 form the voltage-sensing domain, with S5 and S6 along with the pore loops of domains I through IV forming an ion channel [40,41,42,43]. The mutations L925M and L1024V were located in S5 and S6 of domain II, respectively, while mutations F1534S and F1538I were in S6 of domain III (Figure 3D).

### 3.3. Molecular Docking of Tucytb with Bifenazate

**Figure 4:**
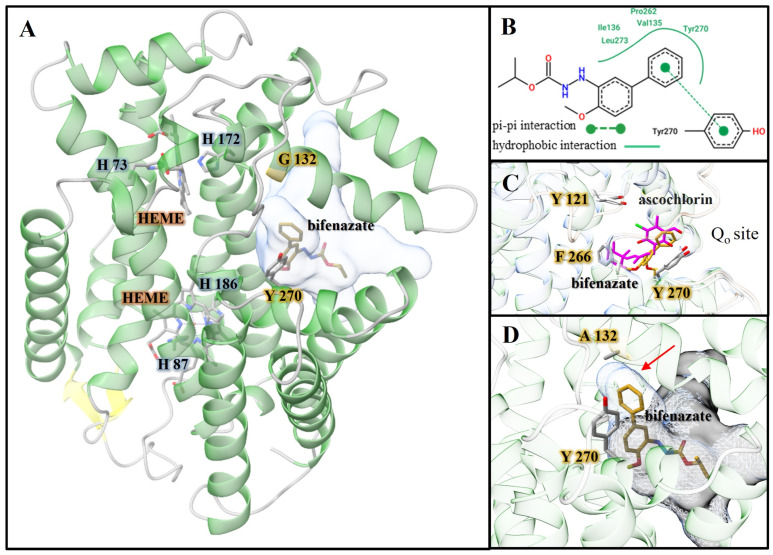
Tucytb AlphaFold2 model and molecular docking results. (**A**) The structure is depicted as a ribbon diagram with a transparent surface overlay of the cavity representing the docked bifenazate binding pocket. The HEME cofactor of cytob is labeled in orange. The top-scoring binding pose of bifenazate (−7.6 kcal/mol) is shown, colored by heteroatom CPK scheme and carbons colored gold. The G132 residue in wt-TuCytb is highlighted in gold. Tyrosine 270, predicted to form an edge-on-face π-π interaction with docked bifenazate, is shown. The two heme cofactors are shown with the side chains of their respective conserved histidine ligands. (**B**) Diagram of predicted binding interactions between TuCytb and docked bifenazate; (**C**) Q_0_ site of chicken cytochrome b (PDB ID 3H1L) superimposed onto TSSM Cytb Alphafold2 model. (**D**) Zoomed-in view of the bifenazate binding pocket of TSSM Cytb A132 mutant; the G132A mutation is predicted to disrupt the key edge-on-face π-π interaction observed in the molecular docking study shown in (**C**). The red arrow highlights extra binding pocket volume present in the wild-type protein but absent in the A132 mutant.

Cytochrome b is part of the larger multimeric protein containing multiple cofactors, all together named the cytochrome bc1 complex or complex III, and it is located within the mitochondria [44,45]. The bc1 complex is a major component of the cellular respiratory chain. A dysfunction in the bc1 complex can lead to disruptions in the electron transport chain and the synthesis of ATP. The closest homologue to Tucytb, with an experimentally determined structure, had a sequence identity of 45.72% and was cytochrome b from the crystal structure of the chicken bc1 complex (PDB ID 3TGU) [45]. When the AlphaFold2 model of Tucytb was superimposed with chicken cytochrome b, the Cα RMSD value was 1.68 Å. The core of the TuCytb model contained a bundle of eight helices. Two heme cofactor molecules were placed in Tucytb based on the superimposed 3TGU crystal structure (Figure 4A), and four conserved heme ligands H73, H172, H87, and H186 were identified. Previous studies have revealed the Qo site of cytochrome b to be a binding pocket for inhibitors [46]. Additionally, it has been shown that bifenazate works by acting as an inhibitor of cytochrome bc1 complex at the Qo site, and that mutations at this site are found in bifenazate-resistant strains of *T. urticae* [47,48]. In this study, the molecular docking of bifenazate with the AlphaFold2 model of Tucytb was performed, and the highest scoring binding pose (−7.6 kcal/mol) placed it in the Qo site of the Tucytb model (Figure 4). Further examination revealed multiple conserved aromatic amino acids in the presumptive binding pocket of Tucytb, including Tyr 270, Phe 266, and Tyr 121. To further examine potential binding interactions, binding pocket cavities were predicted for the wild-type (wt) Tucytb and mutant G132A Tucytb models. For wt-Tucytb, the largest cavity and potential binding pocket was predicted to be adjacent to G132. When the cavity was reanalyzed after calculating it for the mutant G132A model, the pocket was shifted away from the location where bifenazate docked in the wild-type protein, as seen in Figure 4D.

### 3.4. Molecular Docking of TuCHS1 with Etoxazole

**Figure 5:**
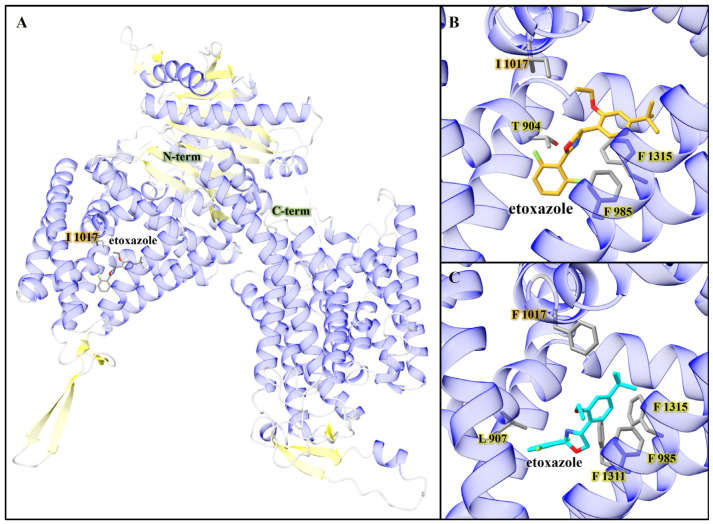
TuCHS1 AlphaFold2 model and molecular docking results. (**A**) TuCHS1 is shown as a ribbon diagram with helices in blue, sheets in yellow, and coils in gray. The highest scoring docked pose of etoxazole adjacent to Ile 1017, in wt-TuCHS1, is shown and colored by atom. (**B**) Zoomed-in view of etoxazole (−8.325 kcal/mol) docked with wt-TuCHS1 model, and colored goldenrod by heteroatom. The side chain of Ile 1017 is shown, along with Phe 1315, which was within the π-π interaction distance of docked etoxazole. Sidechains of Phe 985 and Thr 904 are also shown and were within the hydrophobic interaction distance of etoxazole. (**C**) Zoomed-in view of I1017F mutant TuCHS1 with the highest scoring pose (−8.439 kcal/mol) of the adjacent docked etoxazole to the I1017F mutation. The sidechains of hydrophobic interaction residues are shown (Phe 1311, Phe 985, and Leu 907), along with the sidechain of Phe 1315, which was within the π-π interaction distance of docked etoxazole.

TuCHS1 AlphaFold2 models were constructed for both wt-TuCHS1 and the mutant I1017F, and molecular docking was performed with etoxazole (Figure 5). Favorable binding poses were found in a pocket adjacent to the location of the mutation I1017F. In the wt-TuCHS1 docked etoxazole, favorable hydrophobic interactions were observed between docked etoxazole and residues Phe 985, Phe 1315, Tyr 904, and a face-to-face π-π interaction with Phe 1315. For the I1017F mutant TuCHS1 model, etoxazole docked in the same pocket and displayed hydrophobic interactions with Leu 907, Phe 985, Phe 1311, and Phe 1315. Additionally, there was a π-π interaction with Phe 1315.

## 4. Discussion

This study provides a comprehensive diagnostic analysis of acaricide resistance in TSSM populations from hop and mint fields by integrating bioassays, molecular screening, and structural modeling. By combining bioassays, genetic screening, and structural biology, this study advances diagnostic tools for monitoring acaricide resistance and supports precision pest management strategies for sustainable agricultural systems.

Pyrethroids are among the most widely used insecticides/acaricides globally due to their low cost, effectiveness, and broad-spectrum activity against various pests, including TSSMs [49]. Pyrethroids specifically target VGSCs in the nervous system of insects. By binding to these channels, pyrethroids prevent their normal closure, leading to disrupted nerve impulse propagation. This disruption causes prolonged nerve excitation, which results in the paralysis and eventual death of the insect [50]. Resistance to pyrethroids has been identified in at least nine insect orders and mites, with more than 50 distinct mutations reported associated with pyrethroid resistance [21,51]. While the *kdr* mutation, L1014F, has been identified in certain mites, such as predatory mite *Phytoseiulus persimilis* [52], it is generally absent in other spider mite species. Instead, mutations F1538I + A1215D and L1024V confer significant resistance to bifenthrin, fenpropathrin, and fluvalinate in TSSMs [53]. Additional mutations found in TSSM populations include L925M, F1538I, and the combination F1538I + F1534S [54,55]. In this study, the F1538I mutation was the predominant mutation detected in all TSSM populations from hop and mint fields, except for a single hop TSSM population. This mutation has been functionally characterized and widely associated with pyrethroid resistance in TSSMs and other insect populations [49,50,56]. F1538 is one of several aromatic residues predicted to interact with the alcohol groups of double-ring cyclic pyrethroids, and mutations at this site are expected to destabilize this high-affinity binding, leading to resistance [54]. In addition, the mutation combinations L925M + F1538I, L925M + L1024V + F1538I, and F1534S + F1538I were also detected in hop and mite TSSM populations tested. Mutations at L925 in insect *VGSC*s have previously been documented to alter susceptibility to pyrethroids. For example, L925I found in *Bemisia tabaci para* and L925V in *Varroa destructor* were previously shown to increase resistance to pyrethroids [22,57,58]. Moreover, L1024V (domain II-S6) and F1538I (domain III-S6) in *TuVGSC* have been linked to pyrethroid resistance in *T. urticae* [59]. Additionally, the F1534S mutation located on domain III-S6 has been reported as one of the most common mutations in *Aedes albopictus* and *Aedes aegypti* that confers a low level of resistance to type I pyrethroids, such as bifenthrin [60,61,62]. The detection of these mutations in field-collected populations emphasizes the need for ongoing surveillance and resistance management strategies to mitigate the spread of resistant TSSM populations. Besides target site insensitivity, enhanced metabolic detoxification by cytochrome P450s, carboxyl/cholinesterase, and glutathione S-transferases may also contribute to bifenthrin resistance in TSSMs [8,49].

Bifenazate, a selective hydrazine carbazate acaricide targeting complex III of the mitochondrial electron transport chain, was launched in 1999 and first registered for pest control in Washington in 2002. It is widely used to manage spider mites [4]. Mutations at the Quinol oxidation (Qo) site of the cytochrome b (*Tucytb*) gene are linked to resistance to bifenazate, a complex III inhibitor that targets the mitochondrial electron transport chain, a critical component of cellular respiration in eukaryotes [48]. Specific mutations, including G126S, G132A, G133T, I136T, S141F, L258F, D161G, and P262T, and combinations such as G126S + S141F/A133T/I136T in the conserved cd-1 and ef-helices of Tucytb, have been associated with bifenazate resistance [48,63,64]. In our previous study of TSSM populations in hop fields, only the G126S mutation was detected. However, recent findings indicate that G126S alone does not confer bifenazate resistance, suggesting it is not associated with low to moderate resistance in hop TSSM populations [65]. In this study, instead of G126S, we identified the G132A mutation in the cd1-helix in TSSM populations collected from both hops and mint (Figure 2, Table 2). The G132A mutation, identified in two bifenazate-resistant strains (JP-R and FS1), was confirmed to be maternally inherited through reciprocal crosses, indicating that this mutation alone contributes to bifenazate resistance [64]. From a structural perspective, docking analysis suggests that one or more aromatic residues may play a key role in bifenazate binding at the Qo site through π-π interactions. In our Tucytb model and molecular docking study, Tyr270 specifically formed an edge-on-face π-π interaction with bifenazate (Figure 4B). The structure of chicken cytochrome b (PDB ID: 3H1L) complexed with an inhibitor was superimposed onto the Tucytb model, revealing that the bound inhibitor in 3H1L occupied a position analogous to the docked bifenazate in Tucytb [46] (Figure 4C). Additionally, the aromatic residues Tyr270, Phe266, and Tyr121 in the Qo site were conserved in both proteins. Our molecular docking analysis suggests that the G132A mutation alters the binding pocket of Tucytb, potentially reducing bifenazate affinity. In the wild-type protein, the largest predicted cavity was adjacent to G132, aligning with the bifenazate docking site. However, in the G132A mutant model, the binding pocket was altered in this location (Figure 4D), disrupting the π-π interactions observed in molecular docking. This shift, caused by replacing hydrogen with a methyl group, may be sufficient to displace bifenazate from its preferred binding orientation, thereby reducing its binding affinity to Tucytb. In addition to target site insensitivity, enhanced metabolic detoxification may also play a role in the high level of bifenazate resistance observed in the field [10,63,66]. Future transcriptome analysis and functional studies will provide deeper insights into resistance mechanisms and support diagnostic monitoring efforts.

Mite growth inhibitors, such as etoxazole and hexythiazox, have proven to be valuable IPM tools for TSSM control in both greenhouses and field conditions due to their selective activities against TSSMs compared to predatory mites [67,68,69]. Our previous studies revealed that etoxazole and hexythiazox have been routinely used in hopyards and mint fields since their registration in the 1980s and 2000s, respectively. By 2016, approximately 60% of hop farms in Washington were treated with these acaricides [9,10]. Additionally, cross-resistance had been reported between etoxazole, hexythiazox, bifenazate, and bifenthrin [68]. Population-level bulk segregant analysis combined with high-resolution genetic mapping identified the chitin synthase 1 (*TuCHS1*) gene as the target site for the MGIs etoxazole, clofentezine, and hexythiazox. CHS1, a transmembrane protein involved in chitin deposition, harbors the non-synonymous I1017F mutation, which has been shown to confer resistance to these acaricides [70]. A recent study showed that the I1017F associated with resistance to MGIs was found at high frequencies (80–100%) in all rose glasshouse populations in China [56]. The frequency of the I1017F mutation in TSSM populations from hop fields increased significantly, rising from 25% in 2015 and 88% in 2016 [10,68] to 94% in 2022 and 2023. Moreover, I1017F was not only prevalent in hop TSSM populations but was also detected in 100% of mint TSSM populations, and it was fixed in 33% of populations (Figure 2, Table 2). These findings strongly recommend the cautious use of MGIs in both hops and mint to mitigate further resistance development. The molecular docking of etoxazole with the AlphaFold2-predicted structure of TuCHS1 revealed that the compound binds within a hydrophobic cleft located adjacent to residue I1017. The binding site is in a membrane-proximal region of the protein, adjacent to the predicted chitin translocation channel. The inferred binding location for etoxazole is consistent with findings in previous studies [71]. Notably, docking results suggest that etoxazole does not bind in the active site, indicating that it does not inhibit chitin polymerization directly. Instead, its binding within the transmembrane cleft supports a mechanism involving the obstruction of chitin extrusion. The I1017F mutation associated with acaricide resistance likely disrupts one or more key binding interactions, thereby reducing etoxazole affinity. Additional protein ligand binding assays would be needed to measure any actual change in affinity.

Abamectin (avermectin B1a:B1b = 80:20) is a widely used macrocyclic lactone pesticide for controlling spider mites in hop and mint fields before harvest, due to its short residual effects. Spray records indicate that historically, approximately 98% of hop acreage in Washington was treated with abamectin at least once per year, with 80% receiving as many as three applications annually [9]. The use of abamectin in recent years has dropped substantially due to field control failures. Research has identified mutations in the genes encoding TuGluCls as key contributors to this resistance. For instance, specific mutations such as G314D in *TuGluCl1* and I321T, G326E, V327G, and L329F in *TuGluCl3* have been detected in TSSM populations collected in Europe and China, which may contribute to abamectin resistance [56,72,73,74]. However, no mutations had been identified from hop and mint TSSM populations, suggesting that alternative mechanisms may be involved. Recent studies indicated that the overexpression of cytochrome P450s, glutathione S-transferases, UDP-glycosyltransferases, and cysteine peptidases may be associated with abamectin resistance in TSSM populations [8,72,75]. Future studies should focus on developing diagnostic tools by integrating these molecular markers into resistance monitoring systems.

## 5. Conclusions

Overall, the high resistance levels and frequent resistance-associated mutations identified in this study underscore the need for a comprehensive pest management strategy that incorporates both preventive and corrective measures. Continued research into resistance mechanisms, along with the development of holistic, rapid diagnostic tools and effective control strategies, is essential for sustaining the effectiveness of pesticide applications and ensuring the economic viability of crop production. Our findings provide novel insights into the complex process of TSSM adaptation to various acaricides across different crop systems. By deepening our understanding of resistance mechanisms and exploring alternative control options, we can better protect valuable crops like hops and mint from the economic losses inflicted by this pervasive pest.

## Figures and Tables

**Table 1:** Genetic variations of *TuVGSC* mutations on domains II, III, and II-III. Ph: TSSM population collected from hopyards; Pm: TSSM population collected from mint fields.

Population	*TuVGSC* II						*TuVGSC* III		*TuVGSC* II-III
	M918L	L925M	T929I	L932F	L1014H	L1024V	F1534S	F1538I	A1215D
Susceptible	M	L	T	L	L	L	F	F	A
Ph-1	M	L	T	L	L	L	F	F	A
Ph-2	M	L/M	T	L	L	L	F	F/I	A
Ph-3	M	L/M	T	L	L	L	---	---	A
Ph-4	M	L/M	T	L	L	L	---	---	A
Ph-5	---	---	---	---	---	---	F/S	F/I	A
Ph-6	M	L/M	T	L	L	L	F	F/I	A
Ph-7	M	L/M	T	L	L	L	F	F/I	A
Ph-8	M	L	T	L	L	L	F	F/I	A
Ph-9	M	L/M	T	L	L	L/V	F	F/I	A
Ph-10	M	L	T	L	L	L	F	F/I	A
Ph-11	M	L	T	L	L	L	F	F/I	A
Ph-12	M	L/M	T	L	L	L	F	I	A
Ph-13	M	L	T	L	L	L	F	F/I	A
Ph-14	M	L	T	L	L	L	F	F/I	A
Ph-15	M	L/M	T	L	L	L	F	F/I	A
Ph-16	M	L	T	L	L	L	F	F/I	A
Ph-17	M	L	T	L	L	L	L	F/I	A
Pm-1	M	L	T	L	L	L	F	F/I	A
Pm-2	M	L	T	L	L	L	F	F/I	A
Pm-3	M	L/M	T	L	L	L/V	F/S	F/I	A
Pm-4	M	L/M	T	L	L	L	F	F/I	A
Pm-5	M	L	T	L	L	L	F	F/I	A
Pm-6	M	L	T	L	L	L	F	F/I	A

**Table 2:** Genetic variations of *Tucytb* and *TuCHS 1* mutations. Ph: TSSM population collected from hopyards; Pm: TSSM population collected from mint fields.

Population	*TuCytb*								*TuCHS 1*
	G126S	G132A	A133T	I136T	S141F	L258F	D161G	P262T	I1017F
Susceptible	G	G	A	I	S	L	D	P	I
Ph-1	G	G	A	I	S	L	D	P	I/F
Ph-2	G	G	A	I	S	L	D	P	I/F
Ph-3	---	---	---	---	---	---	---	---	I/F
Ph-4	G	G	A	I	S	L	D	P	I
Ph-5	G	G	A	I	S	L	D	P	I/F
Ph-6	G	G/A	A	I	S	L	D	P	I/F
Ph-7	G	G/A	A	I	S	L	D	P	I/F
Ph-8	G	A	A	I	S	L	D	P	F
Ph-9	G	G/A	A	I	S	L	D	P	F
Ph-10	G	G/A	A	I	S	L	D	P	I/F
Ph-11	G	G/A	A	I	S	L	D	P	F
Ph-12	G	G/A	A	I	S	L	D	P	I/F
Ph-13	G	G/A	A	I	S	L	D	P	I/F
Ph-14	G	G	A	I	S	L	D	P	I/F
Ph-15	G	G/A	A	I	S	L	D	P	F
Ph-16	G	G/A	A	I	S	L	D	P	F
Ph-17	G	G/A	A	I	S	L	D	P	F
Pm-1	G	A	A	I	S	L	D	P	F
Pm-2	G	A	A	I	S	L	D	P	I/F
Pm-3	G	G	A	I	S	L	D	P	I/F
Pm-4	--	--	--	--	S	L	D	P	I/F
Pm-5	G	G	A	I	S	L	D	P	I/F
Pm-6	G	G	A	I	S	L	D	P	F

## Data Availability

The original contributions presented in this study are included in the article/Supplementary Material. Further inquiries can be directed to the corresponding author.

## Supplementary Materials

The following supporting information can be downloaded at https://www.mdpi.com/article/10.3390/insects16050440/s1: Table S1: Information of the susceptible and 23 field collected two-spotted spider mite populations. Table S2: PCR primer information used in this study. Table S3: Acaricide information used in this study. Table S4: The susceptibility of the susceptible and field-collected two-spotted spider mite populations to abamectin and bifenazate.
