# Supplementary material for: Acaricide Resistance Monitoring and Structural Insights for Precision Tetranychus urticae Management
Source: Insects. 2025 Apr 23;16(5):440. doi: 10.3390/insects16050440 (PMC12112526; doi:10.3390/insects16050440)
Supplement: Supplementary file 1 [file insects-16-00440-s001.zip › insects-3518075-supplementary.pdf]

Table S1. Information of the susceptible and 23 field collected two-spotted spider mite populations.

Population	Location		Host plants	Collection Year
	Latitude	Longitude		
Susceptible	---	---	Beans	1995
Ph-1	46.549605	-120.43829	Hops	2022
Ph-2	46.288091	-119.63029	Hops	2022
Ph-3	46.286281	-119.62778	Hops	2022
Ph-4	46.331238	-120.20000	Hops	2022
Ph-5	46.346115	-120.34647	Hops	2022
Ph-6	46.543972	-120.40827	Hops	2023
Ph-7	46.540702	-120.30020	Hops	2023
Ph-8	46.550984	-120.30020	Hops	2023
Ph-9	46.555162	-120.30094	Hops	2023
Ph-10	46.557312	-120.30064	Hops	2023
Ph-11	46.527331	-120.25256	Hops	2023
Ph-12	46.530941	-120.25342	Hops	2023
Ph-13	46.540465	-120.27643	Hops	2023
Ph-14	46.279834	-120.27983	Hops	2023
Ph-15	46.586266	-120.42270	Hops	2023
Ph-16	46.537812	-120.32824	Hops	2023
Ph-17	46.533373	-120.38752	Hops	2023
Pm-1	46.289904	-120.15303	Mint	2023
Pm-2	46.338517	-120.23053	Mint	2023
Pm-3	46.948625	-119.66013	Mint	2023
Pm-4	46.540026	-120.34358	Mint	2023
Pm-5	46.323241	-120.35791	Mint	2023
Pm-6	46.360334	-120.31259	Mint	2023

Note: Ph stands for TSSM population collected from hopyards; Pm stands for TSSM population collected from mint fields.

Table S2. PCR primer information used in this study.

Primer name	Gene name	Gene ID	Primer sequence (5'→ 3')	Amplicon (bp)	Annealing T. (ºC)	Reference
TukdrIIF2<br>TukdrIIR2	voltage-gated sodium channel	JN881331.1	F: GTTCGAGGTCTTTCAGTTTTACG<br>R: GGCTTCTTGAAGTTTCTTGGTG	489	53.9<br>54.4	[9]
TukdrII-IIIF<br>TukdrII-IIIR	voltage-gated sodium channel	JN881331.1	F: TGGACAATTATTATGGACCATGC<br>R: ATGATGGCAGCCAATACACC	255	53.0<br>54.3	[9]
TukdrIIIF1<br>TukdrIIIR2	voltage-gated sodium channel	JN881331.1	F: TGGACAATTATTATGGACCATGC<br>R: GTTTCTTTGATCCCATCTTTTTCAT	259	53.2<br>52.2	[9]
TucytbF2<br>TucytbWTR	cytochrome b (cytb)	EU556749.1	F: AAAACAAATGTGAATTTCAGGG<br>R: TGGTACAGATCGTAGAATTGCG	500	50.1<br>54.6	[48]
TuCHS1F<br>TuCHS1R	Chitin Synthase1	tetur03g08510	F: TCCGCTTGTTATGCACTACTC<br>R: ACCTGAACAAGTTTGCCAGAC	415	54.7<br>55.7	[10]
TuGluCl1F<br>TuGluCl1R	Glutamate –gated chloride channel 1	tetur02g04080	F: TTGGATTGACCCTAACTCAGCAG<br>R: TTGCACCAACAATTCCTTGAGG	270	56.8<br>56.1	[9, 73]
TuGluCl3F2<br>TuGluCl3R2	Glutamate –gated chloride channel 3	tetur10g03090	F: TTGGATCCAAATGCTATTCCTGC<br>R: AACTTGCTGTTGTTGATTTTCTGC	255	55.6<br>55.0	[9, 73]

Table S3. Acaricide information used in this study.

Acaricides	IRAC group	Chemical structure	Target protein	Mutations	Reference
Bifenthrin	3A Sodium channel modulators	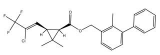	voltage-gated sodium channel (TuVGSC)	M918L, L925M, T929I, L932F, L1014H, L1024V F1534S F1538I, A1215D	[4, 9, 21, 51, 54]
Bifenazate	20D Mitochondrial complex III electron transport inhibitors –Qo site	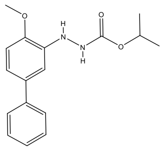	cytochrome b (Tucytb)	G126S, G132A, A133T, I136T, S141F, D161G, L258F, P262T	[48, 64, 66]
Etoxazole	10B Inhibitors of chitin biosynthesis affecting CHS1	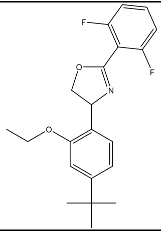	Chitin Synthase 1 (TuCHS 1)	I1017F	[10, 68, 70]
Abamectin	6 Glutamate-gated chloride channel (GluCl) allosteric modulators	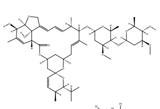 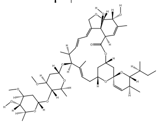	Glutamate –gated chloride channel 1 (TuGluCl1) and Glutamate –gated chloride channel 3 (TuGluCl3)	G314D I321T G326E V327G L329F	[9, 56, 73]

Table S4. The susceptibility of the susceptible and field collected two-spotted spider mite populations to abamectin and bifenazate.

Acaricide	Population	N	LC_{50} (mg a.i./L)	95%CI	Slope ± SEM	RR
Abamectin	Susceptible	4100	0.228	0.12-0.33	1.87± 0.07	1.00
	Ph-1	201	1694.300	-	0.158±0.102	>2000
	Ph-3	200	1108.920	-	0.182±0.105	>2000
	Ph-4	199	749.995	98.57-3.65	0.242±0.113	>2000
	Ph-7	206	36.455	22.89-78.07	0.510±0.097	159.89
	Ph-8	220	11.609	7.43-19.03	0.453±0.077	50.92
	Ph-9	201	11.110	7.69-16.35	0.61±0.088	48.73
	Ph-10	219	46.623	24.44-175.38	0.365±0.083	204.49
	Ph-11	207	51.466	30.34-138.91	0.487±0.101	225.73
	Ph-12	203	55.912	29.81-205.53	0.406±0.092	245.22
	Ph-13	202	193.430	56.49-26645.50	0.282±0.092	848.38
	Ph-14	222	12.428	8.08-20.11	0.475±0.079	54.51
	Ph-15	205	23.094	16.81-34.05	0.725±0.112	101.29
	Ph-17	199	17.627	12.24-27.20	0.608±0.094	77.31
Bifenazate	Susceptible	2195	0.820	0.79-0.85	5.690±0.460	1.00
	Ph-1	204	50.608	15.48-93.82	0.457±0.0934	61.72
	Ph-3	203	68.500	6.63-160.48	0.272±0.079	83.54
	Ph-4	202	14.868	0.58-99.16	0.372±0.029	18.13
	Ph-5	170	29.730	7.80-55.71	0.582±0.128	36.26
	Ph-6	200	26.474	11.15-41.22	0.576±0.125	32.29
	Ph-11	200	22.316	7.82-41.60	0.519±0.069	27.21
	Ph-14	203	14.385	6.57-24.20	0.435±0.079	17.54
	Ph-15	230	168.111	90.78-543.18	0.303±0.092	205.01
	Ph-16	213	260.843	156.13-723.77	0.420±0.099	318.10

Note: Ph stands for TSSM population collected from hopyards; Pm stands for TSSM population collected from mint fields.
